# Different Photosynthetic Response to High Light in Four Triticeae Crops

**DOI:** 10.3390/ijms24021569

**Published:** 2023-01-13

**Authors:** Lun-Xing Chen, Hao-Tian Mao, Shuai Lin, Atta Mohi Ud Din, Xiao-Yan Yin, Ming Yuan, Zhong-Wei Zhang, Shu Yuan, Huai-Yu Zhang, Yang-Er Chen

**Affiliations:** 1 State Key Laboratory of Crop Gene Exploration and Utilization in Southwest China, Sichuan Agricultural University, Chengdu 611130, China; 2 College of Life Science, Sichuan Agricultural University, Ya’an 625014, China; 3 College of Resources, Sichuan Agricultural University, Chengdu 611130, China

**Keywords:** high light, photosynthesis, *Triticea* cereals, chlorophyll fluorescence, thylakoid

## Abstract

Photosynthetic capacity is usually affected by light intensity in the field. In this study, photosynthetic characteristics of four different Triticeae crops (wheat, triticale, barley, and highland barley) were investigated based on chlorophyll fluorescence and the level of photosynthetic proteins under high light. Compared with wheat, three cereals (triticale, barley, and highland barley) presented higher photochemical efficiency and heat dissipation under normal light and high light for 3 h, especially highland barley. In contrast, lower photoinhibition was observed in barley and highland barley relative to wheat and triticale. In addition, barley and highland barley showed a lower decline in D1 and higher increase in Lhcb6 than wheat and triticale under high light. Furthermore, compared with the control, the results obtained from PSII protein phosphorylation showed that the phosphorylation level of PSII reaction center proteins (D1 and D2) was higher in barley and highland barley than that of wheat and triticale. Therefore, we speculated that highland barley can effectively alleviate photodamages to photosynthetic apparatus by high photoprotective dissipation, strong phosphorylation of PSII reaction center proteins, and rapid PSII repair cycle under high light.

## 1. Introduction

On earth, photosynthesis is the most important chemical reaction and is related with crop productivity. In this process, carbon dioxide and water are converted into organic carbohydrates and oxygen is released [1]. In higher plants, photosynthesis occurs in the chloroplasts. Thylakoid membranes of the chloroplasts are mainly composed of photosystem I (PSI), photosystem II (PSII), cytochrome (Cyt) *b_6_f*, and the ATP synthase complex [2]. Among these complexes, PSII is a large pigment−protein complex, which mainly harbors three components: the chlorophyll *a/b* light−harvesting antenna complex (LHC), the oxygen−evolving complex (OEC), and reaction centers (RCs) [3]. However, the photosynthetic machinery, especially PSII, is prone to get limited by environmental stresses such as high light under natural condition.

The growth and development of vascular plants are closely involved in photosynthesis; however, excessive light absorption by pigment molecules usually causes serious oxidative damage to photosynthetic apparatus [4]. High light is one of the most vulnerable environmental stresses in plants and often causes deleterious influences on the growth [5], photosynthetic activity [6], stomatal closure [7], nutritional quality [8], and subsequent yield [7,9], etc. However, plants have evolved a range of physiological mechanisms enabling their photosynthetic apparatus to adapt high light environments [10]. These strategies can be classified into two general categories including short−term and long−term responses to changes in light conditions [11]. The short−term strategies are mainly associated with state transition [12] and nonphotochemical quenching (NPQ) [13]. Long−term light responses mainly include repair system, modulating the abundance of key photosynthetic proteins [14], and regulating expression of photosynthesis−related genes [15].

Triticeae, as a sub−category in Poaceae, is an important tribe in the grass family and contains many important agricultural crops such as wheat, triticale, rye, barley, and highland barley as well as a large number of wild species [16]. Among these Triticeae crops, wheat (*Triticum aestivum* L.) is the most widely planted food crop and mainly grows in temperate regions in the world [17]. Octoploid Triticale (x *Triticosecale* sp.) is a cereal that was intentionally developed by crossing diploid rye and hexaploid wheat in order to combine the positive quality attributes of wheat with durability and hardiness of rye [18]. Barley (*Hordeum vulgare* L.), as one of the earliest domesticated crops of Old World agriculture and the fourth most abundant cereal in the world, is widely used as animal feed, malt, or as a component of various health foods [19]. It has been used as a genetic material for cereal crops in Triticeae [20]. Highland barley (*Hordeum vulgare* Linn. var. *nudum* Hook. f) has been known as hulless barley and the major staple food of Tibetans for generations [19]. Highland barley is mainly cultivated in Qinghai, Tibet, Yunnan, Sichuan, and other plateau areas in China [21]. It is grown at an altitude of 3000–3400 m because of its high drought and cold resistance in the Qinghai−Tibet plateau [19]. A previous study showed that the changes in the sensitivity of PSI photoinhibition depended on wheat cultivars under growth light intensity [22]. Arenas−Corraliza et al. reported that barley showed low light compensation point and high maximum quantum yield relative to wheat under full light conditions [23]. In addition, genome−wide selection footprints from Tibetan hulless barley indicated the adaptive correlation of genes under selection with extensive stressful environmental variables [19]. Although some researches focusing on photosynthesis have been performed in these Triticeae crops, little is known about their photosynthetic acclimation to high light.

It has been known that different plant species probably adopt different mechanisms in response to high light exposure. In this study, we investigated the adaptive responses of four different Triticeae crops (wheat, triticale, barley, and highland barley) to high light by comparing the difference in chlorophyll fluorescence, gas exchange parameters, and the levels of photosynthetic proteins, PSII protein phosphorylation, and thylakoid membrane complexes. Our results suggested that highland barley possesses better photosynthetic capacity and photoprotective mechanisms relative to other three Triticeae crops under high light.

## 2. Results

### 2.1. Effect of High Light on Photosynthetic Pigments

**Figure 1:**
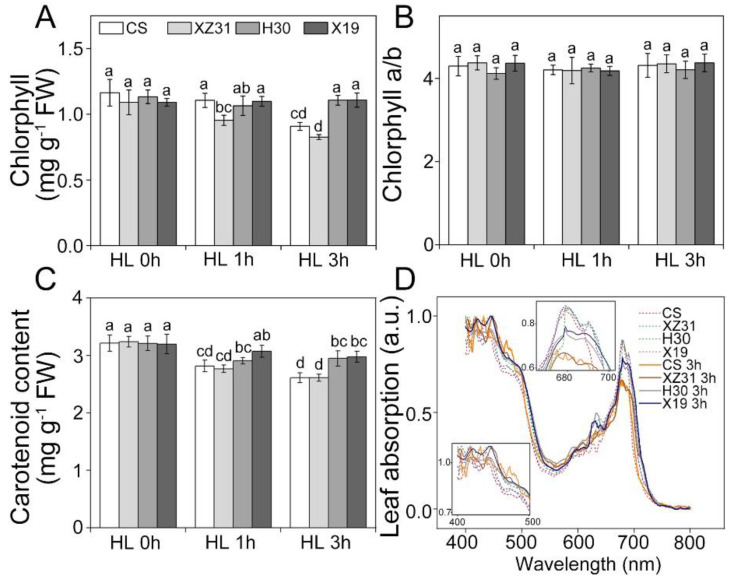
Effects of high light on pigment contents in four Triticeae crops. (**A**) Chlorophyll content. (**B**) Chlorophyll *a/b*. (**C**) Carotenoid content. (**D**) Leaf absorption. Each value shows the means ± SD of three biological replicates (*n* = 3). The different letters indicate significant differences between the treatments (*p* < 0.05) according to Duncan’s multiplication range test. HL 0 h, HL 1 h, and HL 3 h represent high light for 0 h, 1 h, and 3 h, respectively.

The differences in photosynthetic pigment contents among wheat (*Triticuma estivum* L., Chinese Spring, CS), octoploid Triticale (x *Triticosecale* sp., XZ31), barley (*Hordeum vulgare* L., Hua30, H30), and highland barley (*Hordeum vulgare* L. var. nudum Hook. F, Ximalaya 19, X19) were presented in Figure 1. No obvious changes in chlorophyll and carotenoid contents were observed among four Triticeae crops under non−stressful condition (Figure 1A,C). Compared with the control, HL for 3 h resulted in the marked decline in chlorophyll and carotenoid contents in CS and XZ31. Similarly, H30 and X19 showed higher leaf absorption at 400–500 nm and 680 nm under high light for 3 h (Figure 1D).

### 2.2. Effect of High Light on Photosynthetic Efficiency

To compare the photosynthetic capacity of four Triticeae crops, chlorophyll fluorescence and gas exchange parameters were determined in CS, XZ31, H30, and X19 under high light. As shown in Figure S1, X19 showed the highest net photosynthetic rate (*P*_n_), transpiration rate (*T*_r_), and stomatal conductance (*G*_s_) compared to other three Triticeae crops under normal and stress conditions. When compared with the control, intercellular CO_2_ concentration (*C*_i_) significantly decreased in four Triticeae crops exposed to high light for 3 h, especially XZ31 (Figure S1B). Furthermore, 3 h of HL caused the obvious stomata closure in CS, XZ31, H30, and X19 (Figure S2A). Compared with the respective control, H30 and X19 presented the remarkable increase in stomatal perimeter under high light for 3 h (Figure S2B). However, stomatal density did not show obvious change among CS, XZ31, H30, and X19 under high light (Figure S2C).

Considering the differences in gas exchange and stomatal structure, PSI and PSII photochemistry in four plants exposed to high light was further examined. As depicted in Figure S3, X19 showed higher PSI quantum yield (Y(I)) and the quantum yield of non−photochemical energy dissipation of PSI reaction centers due to an acceptor side limitation (Y(ND)) relative to CS under high light for 3 h. In contrast, the quantum yield of non−photochemical energy dissipation in PSI reaction centers due to donor−side limitation (Y(NA)) in CS was higher than that of XZ31, H30, and X19. In addition, the maximal P700 signal (*P*m) was significantly higher in H30 and X19 as compared with CS and XZ31 under normal and stressful conditions (Figure S4). However, the value of the maximum efficiency of PSII photochemistry (Fv/Fm) and effective PSII quantum yield (Y(II)) showed no significant differences among CS, XZ31, H30, and X19 under stressful and non−stressful conditions (Figure S5). Compared with the respective control, 3 h of HL resulted in the significant increase in quantum yield of non−regulated energy dissipation (Y(NO)) of CS and H30. Moreover, a marked decrease in the coefficient of photochemical quenching (qP) was seen in CS under high light for 3 h as compared with the control (Figure S5).

### 2.3. Effect of High Light on Dissipation of Excess Light Energy

**Figure 2:**
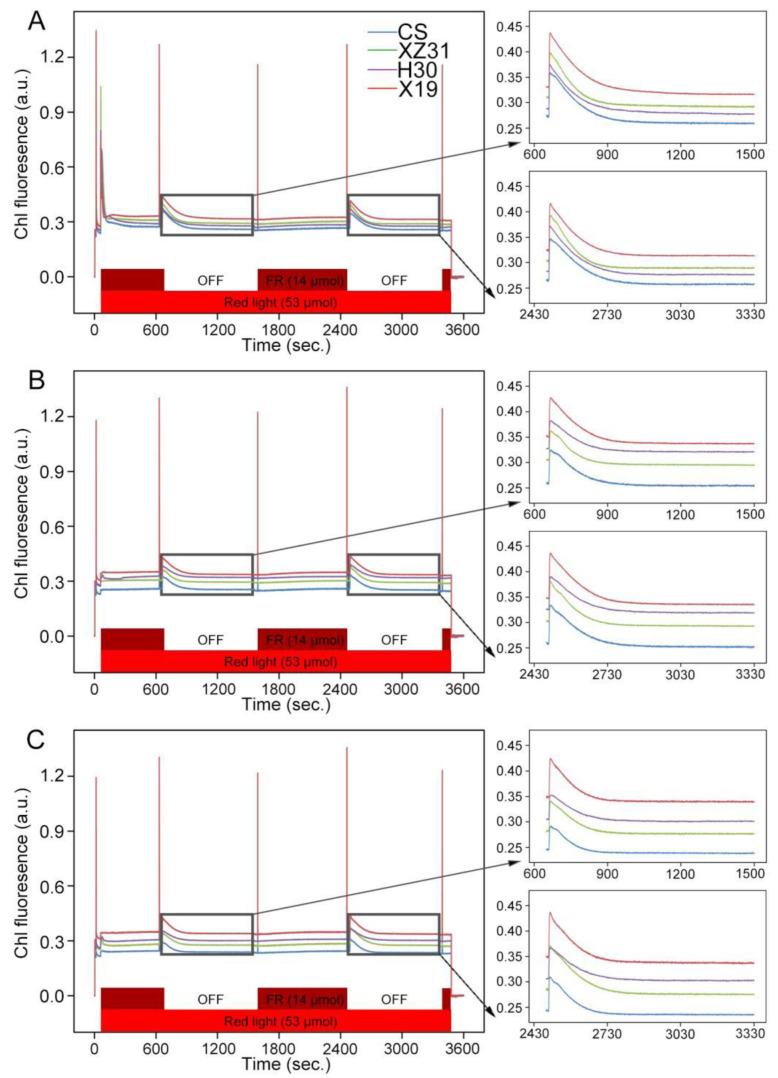
State transitions of four Triticeae crops under high light. Pulse amplitude−modulated fluorescence traces after shifts from state 1 to state 2 light and back. The bars at the bottom indicate illumination with red (shown in red) and far−red (dark red) light. Fluorescence is shown in arbitrary units. (**A**) High light for 0 h. (**B**) High light for 1 h. (**C**) High light for 3 h.

**Figure 3:**
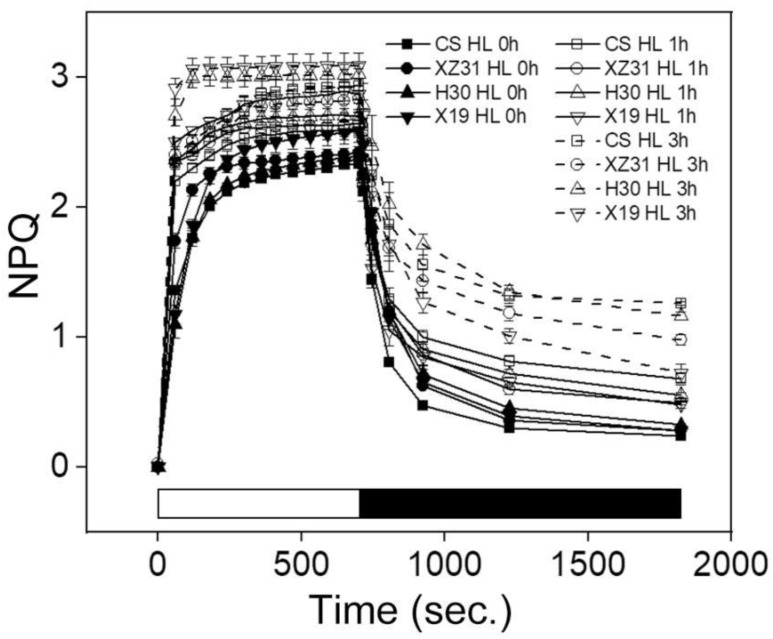
NPQ kinetics of four Triticeae crops under high light. Bars on bottom, white bar (light on) and black bar (dark). The data show the means ± SD of three biological replicates (*n* = 3). HL 0 h, HL 1 h, and HL 3 h represent high light for 0 h, 1 h, and 3 h, respectively.

To explore the change in energy dissipation among four Triticeae crops, nonphotochemical quenching (NPQ) and state transition were determined under high light. Compared with CS, other three Triticeae crops (XZ31, H30, and X19) showed higher transitory increase in fluorescence when the far−red light was turned off under high light for 1 h and 3 h, especially X19 (Figure 2). In addition, the NPQ was significantly upregulated in X19 compared with the other three plants under normal conditions (Figure 3). However, the kinetics of dark relaxation showed no obvious differences among four Triticeae crops. Similarly, the highest NPQ and the fastest dark recovery were observed in X19 as compared with other three plants under high light for 3 h (Figure 3). These results suggested that X19 could effectively dissipate excess light energy under high light.

### 2.4. Effect of High Light on OJIP and Oxygen−Evolution Activity

**Figure 4:**
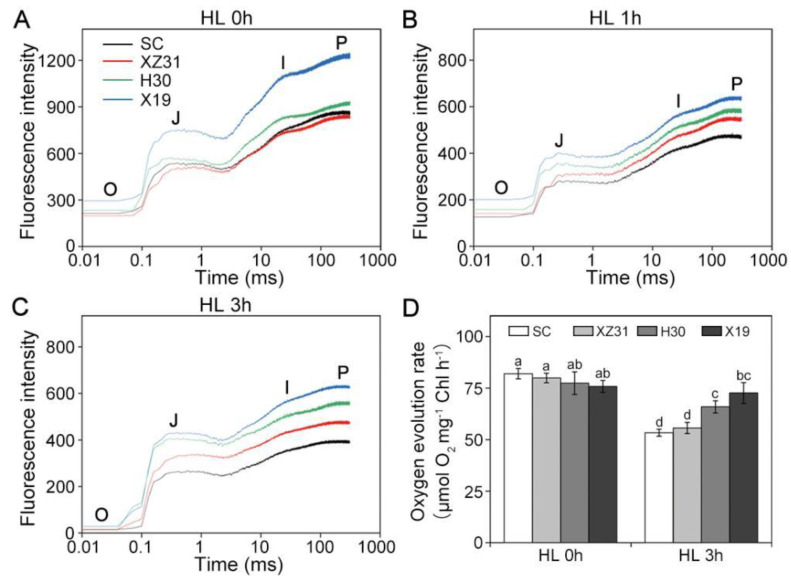
Effects of high light on OJIP transient curves (**A**–**C**) and oxygen evolution (**D**) in four Triticeae crops. Each step shown in the figure indicates the minimal fluorescence intensity when all photosystem II (PSII) reaction centers (RCs) are open (the O step), the intensity at 2 ms (the J step), the intensity at 30 ms (the I step), and the maximal intensity when all PSII RCs are closed (the P step). Each value shows the mean ± SD of three biological replicates (*n* = 3). The different letters indicate significant differences between the treatments (*p* < 0.05) according to Duncan’s multiplication range test. HL 0 h, HL 1 h, and HL 3 h represent high light for 0 h, 1 h, and 3 h, respectively.

The changes in photosynthetic electron transfer chain were observed by OJIP in four Triticeae crops under high light (Figure 4A–C). In OJIP curves of the leaves, each step exhibited a similar response to high light. Among four plants, X19 and CS presented the highest and lowest fluorescence intensity of the O, J, I, and P step under stress and non−stressful conditions (Figure S6). Moreover, OJIP curves in four plants were standardized by *V*_O−P_ under high light (Figure S7). The results showed that the relative variable fluorescence *V*_J_ at 2 ms of the *V*_O−P_ curve increased significantly in X13 and H30 compared with CS and XZ31 under high light for 3 h.

In addition, the rate of steady−state oxygen evolution from isolated thylakoid was determined in four plants under high light (Figure 4D). The capacity of O_2_ evolution showed no significant differences among CS, XZ31, H30, and X19 under non−stress condition, whereas 3 h of HL resulted in the highest decrease in the levels of O_2_ evolution in CS and XZ31 relative to the respective control. Thus, these results indicated that the efficiency of electron transfer was more effective in X19 under high light.

### 2.5. PSII Photoinhibition Analysis

**Figure 5:**
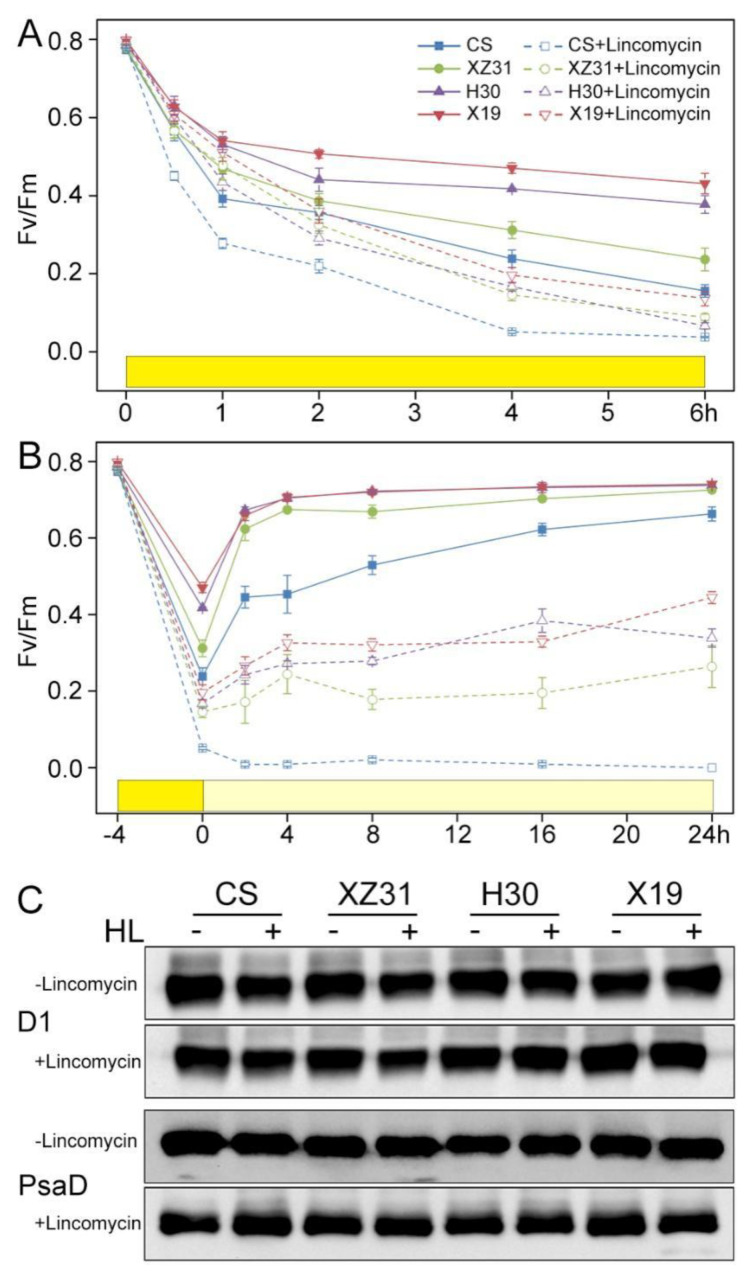
PSII photosensitivity of four Triticeae crops during high light illumination. (**A**) Lincomycin−treated (+) and untreated (−) detached leaves were subjected to high light (1000 µmol photons m^−2^ s^−1^) for 6 h. (**B**) Photoinhibited leaves were recovered at low light intensity (10 µmol photons m^−2^ s^−1^) up to 24 h. Value shows the means ± SD of three biological replicates (*n* = 3). (**C**) Immunoblot analysis of thylakoid proteins using D1 and PsaD antibodies before (−) and after (+) photoinhibition under high light. PsaD was as a loading control.

The sensitivity of PSII to photoinhibition with or without lincomycin was compared among four plants exposed to high light for 6 h (Figure 5). PSII repair was blocked by lincomycin by inhibiting chloroplast protein synthesis. As shown in Figure 5A, the value of Fv/Fm in X19 was significantly higher than that of other three plants in the absence or presence of lincomycin during 6 h of high light. Similarly, X19 and H30 presented the faster rates of recovery from photoinhibition than CS and XZ31 after 4 h of HL (Figure 5B). In addition, the accumulation of PSII reaction center D1 protein in the leaves of four plants was analyzed under high light (Figure 5C). The amount of D1 protein in X19 and H30 was significantly higher than that of CS and XZ31 after 3 h of illumination in the presence of lincomycin. However, the abundance of PSI protein PsaD did not show obvious differences among four plants under high light.

### 2.6. Effect of High Light on Photosynthetic Proteins, Protein Phosphorylation and Thylakoid Membrane Complexes

**Figure 6:**
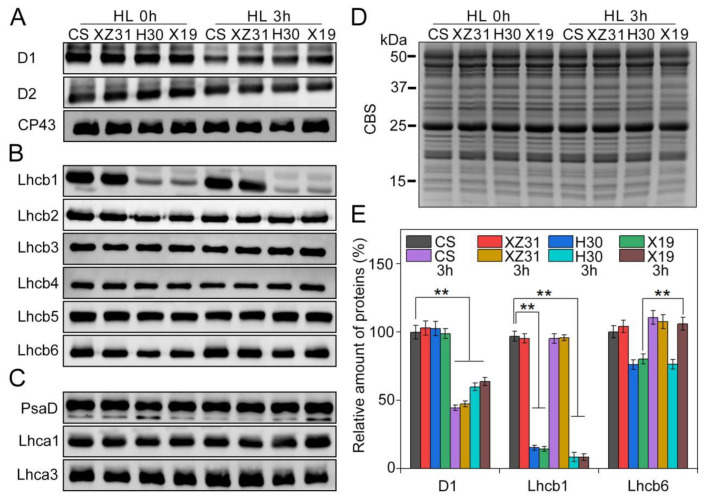
Immunoblot analyses of thylakoid proteins in four Triticeae crops under high light. (**A**) D1, D2, and CP43. (**B**) Lhcb1, Lhcb2, Lhcb3, Lhcb4, Lhcb5, and Lhcb6. (**C**) PsaD, Lhca1, Lhca3. (**D**) Coomassie blue staining (CBS) of SDS−PAGE. (**E**) Quantitative data for D1, Lhcb1, and Lhcb6 proteins. Results are presented relative to the amount of the relative control (100%). ** indicate statistically significant differences at *p* < 0.01 levels by Duncan’s multiple comparison test. 1 μg of total chlorophyll was added to each well. HL 0 h and HL 3 h represent high light for 0 h and 3 h, respectively.

**Figure 7:**
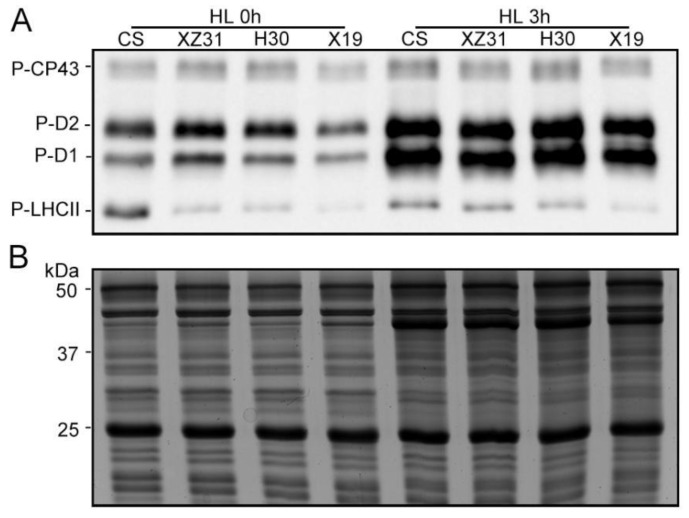
Phosphorylation of thylakoid membrane proteins under high light. (**A**) Immunoblotting of thylakoid proteins from four Triticeae crops was carried out using antiphosphothreonine antibodies. (**B**) Coomassie blue staining (CBS) of SDS−PAGE. HL 0 h and HL 3 h represent high light for 0 h and 3 h, respectively.

**Figure 8:**
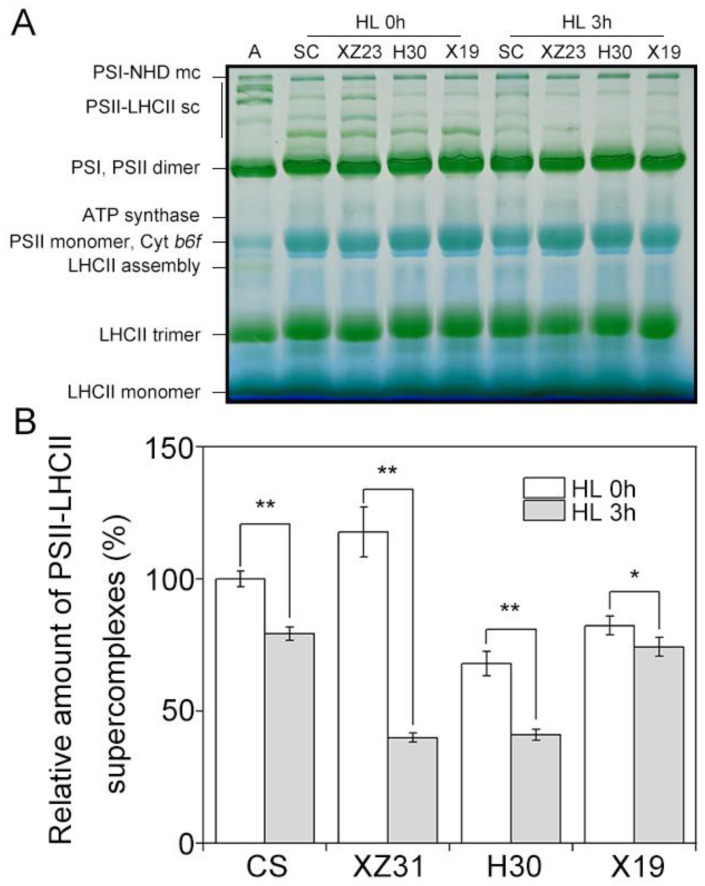
Analysis of thylakoid membrane protein complexes in four Triticeae crops under high light. (**A**) BN−PAGE of thylakoid membranes was performed with 5−12.5% acrylamide after solubilization using 1% (*w*/*v*) DM. (**B**) Quantification of PSII−LHCII supercomplexes. Results are showed relative to the amount of untreated wheat (100%). * and ** indicate statistically significant differences at *p* < 0.05 and *p* < 0.01 levels by Duncan’s multiple comparison test, respectively. HL 0 h and HL 3 h represent high light for 0 h and 3 h, respectively.

To explore the different responses of photosynthetic proteins to high light in four Triticeae crops, thylakoid membrane proteins were analyzed by Western blotting. The levels of almost all analyzed thylakoid membrane proteins showed no obvious differences among four plants except for D1, Lhcb1, and Lhcb6 (Figure 6). When compared with the control, 3 h of HL led to the remarkable decrease in the amount of D1 in four plants, especially CS and XZ31 (Figure 6A,E). It is worth noting that the level of Lhcb1 protein in CS and XZ31 was significantly higher than H30 and X19 under stressful and non−stressful conditions (Figure 6B,E). Moreover, Lhcb6 presented a significant increase in X19 under high light for 3 h relative to the respective control (Figure 6B,E). In addition, the levels of PSII protein phosphorylation were further measured in four plants exposed to high light for 3 h. As depicted in Figure 7, the phosphorylation of four PSII proteins (CP43, D1, D2, and LHCII) was the lowest in X19 compared with CS, XZ31, and H30 under non−stress conditions. Compared with the respective control, X19 presented higher phosphorylation levels of D1 and D2 relative to other three plants under high light for 3 h (Figure S8). However, the accumulation of phosphorylated−LHCII was the lowest in X19 under stressful and non−stressful conditions. Next, thylakoid membrane complexes were also compared between four plants under high light. As reported in Figure 8, the amount of PSII−LHCII super complexes in CS and XZ31 was higher than that of H30 and X19 under non−stress condition. When compared with the control, 3 h of HL caused the significant decline in the amount of PSII−LHCII super complexes in four plants, especially XZ31 and H30.

## 3. Discussion

High intensity light has been thought to be a major limiting factor in photosynthesis of plants under natural environmental conditions. Many research have showed that vascular plants have various photosynthetic acclimation to challenging environmental stimuli to reduce ROS accumulation and subsequent oxidative damage in chloroplasts [5,22,24]. Wheat, triticale, barley, and highland barley are planted widely in different regions of China. Due to the complexity of genetic background, they probably have different physiological responses to environmental stresses [25,26,27]. Here, we compared the photosynthetic performance among four Triticeae crops (wheat, triticale, barley, and highland barley) under high light.

Levels of photosynthetic pigments are closely related to the photosynthetic capacity in higher plants under environmental stresses [22,28,29]. Previous studies have indicated that high chlorophyll content can absorb more light energy and thus result in high photosynthetic ability [30]. However, too much light energy can damage the photosystem [30]. Our previous works found that high light led to an obvious decrease in photosynthetic pigment contents in wheat and *Arabidopsis* plants [6,31]. In accordance with these findings, high light resulted in a significant decline in chlorophyll and carotenoid contents of wheat and triticale, suggesting that barley and highland barley should possess better photosynthetic activity under high light [6]. 

Under environmental stresses, the photosynthetic ability is usually affected by stomatal behavior in higher plants. Many studies indicated that abiotic stresses resulted in a decline in *P*_n_, *T*_r_, and *G*_s_, and an increase in *C*_i_ [31,32,33]. Here, highland barley showed high *P*_n_, *T*_r_, *G*_s_, and *C*_i_ relative to the other three plants, indicating that gas exchange was more effective in highland barley under high light exposure. These results were further confirmed by the data obtained from the structure of stomata under high light. 

Chlorophyll fluorescence has been used to evaluate the photosynthetic efficiency in different plants under stress and non−stress conditions [5,24,34,35]. Our previous work showed that drought−resistant wheat had higher PSII photochemistry than drought−susceptible wheat [24]. In addition, many studies have reported that PSI and PSII activities markedly decreased in some plants exposed to long−time high irradiance [23,35]. In this experiment, we found that X19 and H30 showed high levels of Y(II), qP, Y(NA), and Pm under high light, especially X19, indicating that it possessed more higher photosynthetic capacity in response to high light relative to other three crops. The reason was probably due to the long photosynthetic acclimation to high light or natural fluctuating light conditions [36]. These results were further identified by the data from state transitions and NPQ kinetics (Figure 2 and Figure 3), which are thought to be the key protective mechanisms in response to environmental stresses through the excitation balance between PSI and PSII [30,37] and the dissipation of excess light energy in PSII [38], respectively. A recent study showed that NPQ obviously improved in response to a short duration of high light [39]. Here, our results showed that the rapid rising and the dark recovery in the level of NPQ were observed in X19 compared with other three plants, meaning that highland barley possessed fast dissipation mechanism against excess light energy and high photochemical efficiency under high light [40]. Similarly, the significant upregulation of the fluorescence was seen in H30 and X19 under high light when far−red light was off, specially X19. This reason might be because LHCII migration through protein reversible phosphorylation was more rapid in barley and highland barley under high light.

In addition, the degree of oxidative damage to photosynthetic apparatus can be effectively measured by OJIP under stress condition [41]. In the present study, high light resulted in the significant decline in OJIP parameters, which are usually used in energy absorption and electron transport in PSI and PSII in plants [42]. However, CS and XZ31 showed more obvious decrease in OJIP parameters relative to H30 and X19, indicating that PSII reaction center (RC) and electron transport flux were more effective in barley and highland barley under high light. The results were further verified by the data from photoinhibition, which is an imbalance between the rate of PSII repair and its damage [43]. Under high light, the main reason for photoinhibition is probably owing to the suppression of PSII repair [44]. Here, Fv/Fm value in CS and XZ31 was obviously lower than that of H30 and X19 in the presence and absence of lincomycin during high light for 6 h, meaning that the inhibition of PSII repair was weak in barley and highland barley. It might be because of effective photoprotective mechanism and reactive oxygen species (ROS)−scavenging system under high light [31]. This is similar to the results previously reported in monocotyledons that Fv/Fm decreased significantly in the presence of lincomycin and the recovery process of PSII was dampened under high light [45]. Furthermore, photoinhibition is accompanied with the damage to D1 protein during PSII repair cycle under environmental stresses [46]. In this experiment, we found that high light led to the great decline in the level of D1 with or without lincomycin in wheat and triticale. This reason was probably because the degradation of D1 protein and subsequent repair of PSII RC were more rapid in barley and highland barley under high light.

Many studies have indicated that PSII reaction center protein D1 is the primary target of the photoinduced PSII damage under environmental stresses [31,39]. The main photodamage maintains an oxidative damage to D1 protein and a conformational change [47]. Our previous works found that high light markedly reduced the level of D1 in plants [6,31]. Here, our experiments showed that the amount of D1 protein decreased significantly in four Triticeae crops relative to the control under high light, especially wheat and triticale, suggesting that high light caused a more severe damage to PSII RCs in wheat and triticale. The decrease in the levels of D1 protein was similar with the results from the photoinhibition. Furthermore, the amount of Lhcb1 protein in barley and highland barley was obviously lower than that of wheat and triticale under high light and normal condition. The reason is most likely that the reactivity of antibody is different in different land plants. Lhcb1, as one of the light−harvesting chlorophyll *a/b* binding proteins, is the most abundant protein in eukaryotic phototrophs and is encoded by five genes in *Arabidopsis thaliana* [48]. In addition, Lhcb1 presents an extraordinary degree of evolutionary conservation and is thought to be important for thylakoid structure flexibility [49,50]. A previous report from *Arabidopsis thaliana* showed that the decline in Lhcb1 had partially compensatory upregulation in the amounts of Lhcb2 and Lhcb3 [49]. In addition, Bielczynski et al. reported that the reduction of Lhcb1 and Lhcb2 has no negative effect on the function of photosynthetic devices [51]. In our experiments, the levels of Lhcb2 and Lhcb3 were unchanged in the four plants under high light. Lhcb6 is one of the main component of the light−harvesting antenna proteins and plays an important role in light energy dissipation [52]. Previous research indicated that Lchb5 and Lhcb6 proteins dramatically elevated to mitigate photooxidative damage to PSII under environmental stresses [6,53]. Here, a more significant increase in the amount of Lhcb6 was seen in highland barley relative to the respective control under high light, meaning that more excess energy could be effectively dissipated.

It has been well−known that phosphorylation of PSII−LHCII proteins is usually thought to play important regulatory roles in the PSII repair cycle, the lateral movement of PSII−LHCII, and subsequent energy balance between PSII and PSI [47]. In the present study, high light induced the strong phosphorylation of D1 and D2 proteins in four plants, but the highest increase in their phosphorylation was observed in highland barley compared with the control, implying that the degradation of damaged proteins of PSII RCs with insertion of new synthetic proteins into PSII was more rapid in highland barley [54]. Previous reports have also shown that the phosphorylation of PSII proteins can help plants respond to different environment stresses [27]. Here, our results showed that more obvious phosphorylation of LCHII was found in triticale, barley, and highland barley relative to the control under high light, suggesting that they effectively regulated the distribution of excitation energy between PSI and PSII. In addition, only PSII−LHCII super complexes significantly reduced in four plants under high light, while the most obvious decline in PSII−LHCII super complexes was found in triticale and barley. It was probably due to their high LHCII phosphorylation under high light, which is involved in partial destacking of the grana and movement of PSII−LHCII complexes toward the granna margins [55]. 

## 4. Material and Methods

### 4.1. Plant Materials and Treatments

Seeds of wheat (*Triticuma estivum* L., Chinese Spring, CS), octoploid Triticale (x *Triticosecale* sp., XZ31), barley (*Hordeum vulgare* L., Hua30, H30), and highland barley (*Hordeum vulgare* L. var. nudum Hook. F, Ximalaya 19, X19) were disinfected with 2% sodium hypochlorite (NaClO), washed with deionized water, and germinated in a Petri−dish with wet filter paper for 48 h at 25 °C in the dark. The germinated seedlings were cultured in sterilized sand with 1/2 Hoagland nutrient solution in a climate chamber (25 ± 1 °C) under illumination with 250 μmol photons m^−2^ s^−1^ at the 16/8 h light/dark photoperiod for two weeks. Then, nine seedlings were subjected to high light (1200 μmol photons m^−2^ s^−1^) for 0 h (HL 0 h), 1 h (HL 1 h), and 3 h (HL 3 h) in the greenhouse. After the treatments, the second leaves from the top were used for the following measurements.

### 4.2. Determination of Pigment and Leaf Absorption

Carotenoid and chlorophyll of fresh leaves were extracted with 80% (*v*/*v*) acetone and then measured with a UV−visible spectrophotometer (Hitachi−U2000, Hitachi, Ltd., Tokyo, Japan) according to the previous method [56]. UV−Vis spectrophotometer with a mounted 126 integrating sphere (BioMate 3S, Thermo Fisher Scientific Inc., MA, USA) was used to determine leaf absorption as described previously [51].

### 4.3. Measurements of Gas Exchange and Stomatal Status 

Net photosynthetic rate (*P*_n_), intercellular CO_2_ concentration (*C*_i_), transpiration rate (*T*_r_), and stomatal conductance (*G*_s_) of the intact leaves (2nd leaf) were estimated with the GSF−3000 photosynthesis system (Heinz−Walz Instruments, Effeltrich, Germany). The measuring conditions were illumination of 1000 µmol photon m^−2^ s^−1^, 360 µmol mol^−1^ CO_2_ concentration, and 60−80% relative humidity at 25 °C. 

Stomatal apertures of the second leaves with similar size were assayed as described previously [57]. After HL treatments, leaf lower epidermis cells were stuck using adhesive plastic tapes and then the epidermal strips were removed. A fluorescence microscope (Bx53 System, Olympus Corporation, Tokyo, Japan) was used to examine the stomatal structure.

### 4.4. OJIP and Oxygen−Evolving Activity

The fast phase of Chl *a* fluorescence induction (FI) was determined with a dual PAM−100 fluorometer (Heinz−Walz Instruments, Effeltrich, Germany) according to the method of Lin et al. [35]. To measure OJIP transients, the plants were kept in the dark for at least 30 min, and then the adaxial surface of the leaves was treated with a saturated pulse intensity (5000 µmol photons m^−2^ s^−1^) for 0.5 s. The O, J, I, and P steps were the minimal fluorescence intensity when all the reaction centers (RCs) of PSII are open, the intensity at 2 ms, the intensity at 30 ms, and the maximal intensity when all the RCs of PSII are closed, respectively. To obtain *V*_O−P_ curves, OJIP curves were standardized by O−P as described previously [58].

A Clark−type electrode (Hansatech Instruments, Norfolk, United Kingdom) was used to measure the oxygen−evolving activity of thylakoid membranes obtained from the leaves as the previous method [59]. The assay buffer contained 25 mM Hepes (pH 7.6), the artificial electron acceptor phenyl−*p*−benzoquinone (PpBQ, 0.25 mM), 0.2 M sucrose, 5 mM CaCl_2_, and 10 mM NaCl at 20 °C.

### 4.5. Chlorophyll Fluorescence, NPQ Kinetic, State Transition, and P700 Parameters

Chlorophyll fluorescence was analyzed using an imaging PAM M−series chlorophyll fluorometer (Heinz−Walz Instruments, Effeltrich, Germany) based on our previous method [6]. Prior to the measurements, the samples were kept in the dark for 1 h. The actinic light intensity and the saturation pulse intensity were set to 150 μmol m^−2^ s^−1^ and 8000 μmol m^−2^ s^−1^, respectively. Then, the maximum efficiency of PSII photochemistry (Fv/Fm), effective PSII quantum yield (Y(II)), quantum yield of non−regulated energy dissipation (Y(NO)), and coefficient of photochemical quenching (qP) were imaged and calculated (Maxwell and Johnson, 2000).

Dual PAM−100 fluorometer (Heinz−Walz Instruments, Effeltrich, Germany) was used to analyze state transition and NPQ kinetics of the plants following established protocols [60]. The samples were adapted in the dark for 1 h before the measurements. The NPQ kinetics were calculated according to the Fm value obtained from an untreated sample. At the end of each state transition cycle, Fm′ (Fm level in State I) and Fm″ (Fm level in State II) were recorded by the application of the saturating light pulse.

The redox state of P700 was determined with a Dual PAM−100 fluorometer following the manufacturer’s instructions. After 30 min of adaptation in the dark, the P700 parameters including the photochemical quantum yield of PSI (ΦPSI), the reduction status of PSI receptor side (ΦNA), and the oxidation status of PSI donor side (ΦNA) were calculated as described previously [61].

### 4.6. Photoinhibition and Recovery

The sensitivity of PSII to high light was determined based on the changes in Fv/Fm in the intact leaves of the control and 3 h of HL plants with or without lincomycin [62]. To measure PSII recovery after photoinactivation, the samples were exposed to 1000 µmol photons m^−2^ s^−1^ for 4 h followed by low light (10 µmol photons m^−2^ s^−1^) for 24 h. The Dual PAM−100 fluorometer was used to determine Fv/Fm at a regular time. To analyze D1 damage during photoinhibition, detection of D1 protein was performed by immunoblotting with specific D1 antibody.

### 4.7. SDS−PAGE and Immunoblot Analysis

Thylakoid proteins were extracted from the leaves in the presence of 10 mM NaF under dim light [59], and then were separated by SDS−PAGE (6% acrylamide stacking gel + 15% separation gel + 6 M urea) based on equal chlorophyll. Proteins from the gel were subsequently transferred to polyvinylidene fluoride (PVDF) membrane (Immobilone, MilliPore, Darmstadt, Germany) using standard methods, and then were detected using specific antibodies including Lhca1, Lhca3, PsaD, CP43, D1, D2, Lhcb1, Lhcb2, Lhcb3, Lhcb4, Lhcb5, and Lhcb6 (Agrisera, Umea, Sweden). Phosphorylation of thylakoid proteins was detected by anti−phosphothreonine antibody (Cell Signaling, Ipswich, MA, USA). The immunoblotting signals of proteins were detected using horseradish peroxidase−conjugated anti−rabbit antibody (Agrisera Comp., Umea, Sweden) and ECL reagents (GE Healthcare, Buckinghamshire, UK). Quantity one software (v4.4, Bio−Rad Comp., Hercules, CA, USA) was used to quantify signal amplitude.

### 4.8. Blue−Native PAGE

Blue native−polyacrylamide gel electrophoresis (BN−PAGE) was carried out as previously described by Chen et al. [59]. 1% (*w*/*v*) *n*−dodecyl−*β*−D−maltoside was used to solubilize thylakoid membranes (20 μg Chl) for 10 min in the dark on ice. After centrifugation (18,000× *g*, 20 min) at 4 °C, BN−PAGE was done using a gradient of 5–12.5% acrylamide in the separation gel and a gradually increasing voltage (75–200 V) for 4 h at 4 °C.

### 4.9. Statistics Analysis

The data were analyzed by Duncan’s multiple range tests using SPSS19.0 (IBM, Chicago, IL, USA). All results were expressed as the mean ± standard deviation (SD) of at least three independent replicates. Different lowercase letters in the figures indicate to be significant differences among treatments when *p* < 0.05.

## 5. Conclusions

In the present study, we found that four Triticeae crops (wheat, triticale, barley, and highland barley) showed obviously different photosynthetic acclimation to high light. Our results indicated that highland barley and wheat exhibited the highest and lowest photosynthetic capacity and light energy dissipation under high light, respectively. In highland barley, the effective photosynthetic acclimation to high light is mainly involved in the rapid repair cycle of PSII and strong phosphorylation of PSII RC proteins. Taken together, we propose that highland barley possesses better photoprotective mechanisms under environmental stresses and should be more suitable for planting in some regions with unfavorable environments.

## Data Availability

All information can be downloaded at Supplementary Materials.

## Supplementary Materials

The following supporting information can be downloaded at: https://www.mdpi.com/article/10.3390/ijms24021569/s1.
