# Supplementary material for: Different Photosynthetic Response to High Light in Four Triticeae Crops
Source: Int J Mol Sci. 2023 Jan 13;24(2):1569. doi: 10.3390/ijms24021569 (PMC9862584; doi:10.3390/ijms24021569)
Supplement: Supplementary file 1 [file ijms-24-01569-s001.zip › ijms-2059565-supplementary.pdf]

Different Photosynthetic Response to High Light in Four Triticeae Crops

Lun-Xing Chen [1,2,†], Hao-Tian Mao [2,†], Shuai Lin [2], Atta Mohi Ud Din [2], Xiao-Yan Yin [2], Ming Yuan [2], Zhong-Wei Zhang [3], Shu Yuan [3], Huai-Yu Zhang [2] and Yang-Er Chen [1,2,*]

[1] State Key Laboratory of Crop Gene Exploration and Utilization in Southwest China, Sichuan Agricultural University, Chengdu 611130, China

[2] College of Life Science, Sichuan Agricultural University, Ya'an 625014, China

[3] College of Resources, Sichuan Agricultural University, Chengdu 611130, China

[*] Correspondence: chenyanger@sicau.edu.cn; Tel.: +86-835-2886653

[†] These authors contributed equally to this study.

Supplementary Figures S1–S8

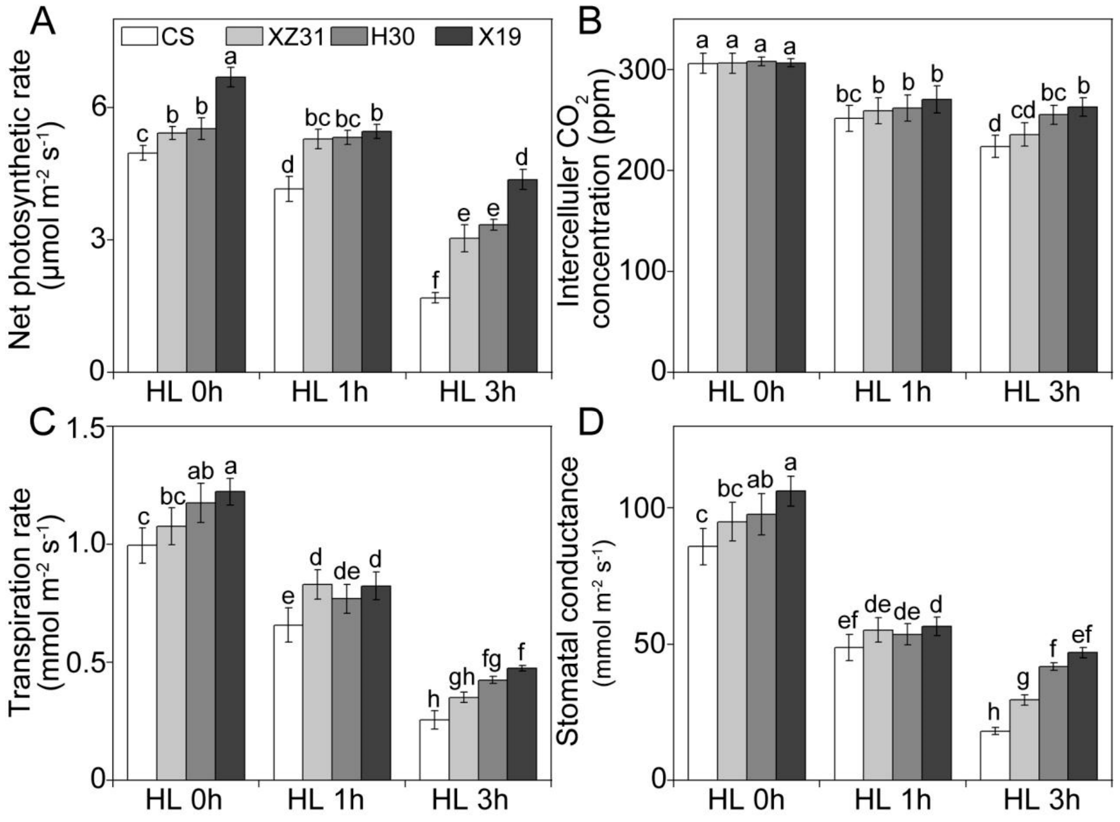

Figure S1. Effects of high light on gas exchange parameters in four Triticeae crops. (A) Net photosynthetic rate. (B) Intercellular CO_2 concentration. (C) Transpiration rate. (D) stomatal conductance. Each value shows the means ± SD of three biological replicates. The different letters indicate significant differences between the treatments ($p < 0.05$) according to Duncan's multiplication range test. HL 0h, HL 1h, and HL 3h represent high light for 0 h, 1 h, and 3 h, respectively.

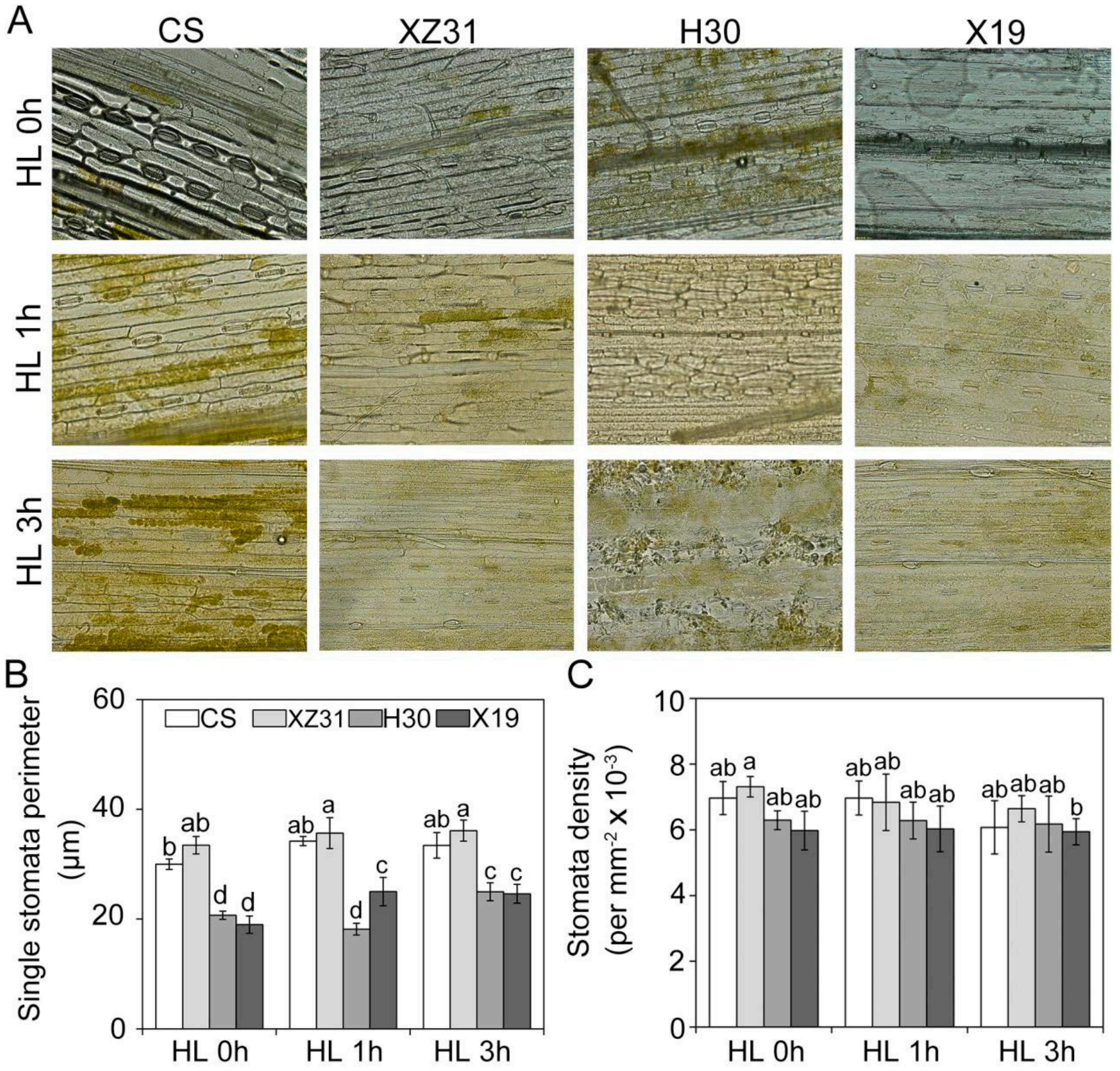

Figure S2. Analysis of stomatal characteristics of four Triticeae crops under high light. (A) Stomatal status. (B) Single stomatal perimeter. (C) Stomata density. Each value shows the means ± SD of three biological replicates. The different letters indicate significant differences between the treatments (p < 0.05) according to Duncan's multiplication range test. HL 0h, HL 1h, and HL 3h represent high light for 0 h, 1 h, and 3 h, respectively.

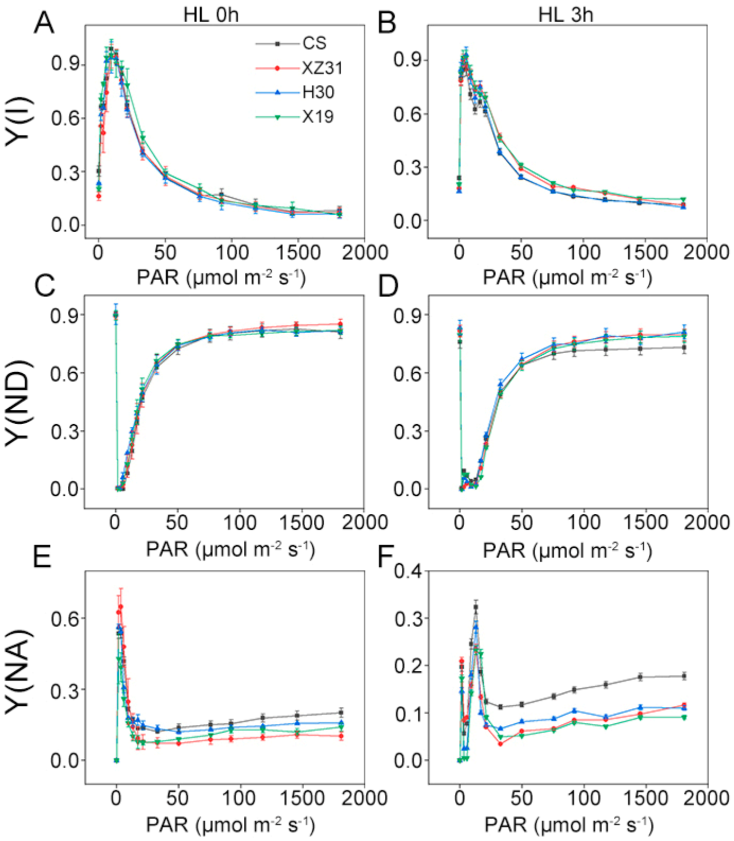

Figure S3. Effects of high light on PSI photochemistry in four Triticeae crops. (A and B) Y(I), effective quantum yield of PSI. (C and D) Y(ND), quantum yield of non-photochemical energy dissipation in PSI reaction centers due to donor side limitation. (E and F) Y(NA), quantum yield of non-photochemical energy dissipation of PSI reaction centers due to acceptor side limitation. HL 0h and HL 3h represent high light for 0 h and 3 h, respectively.

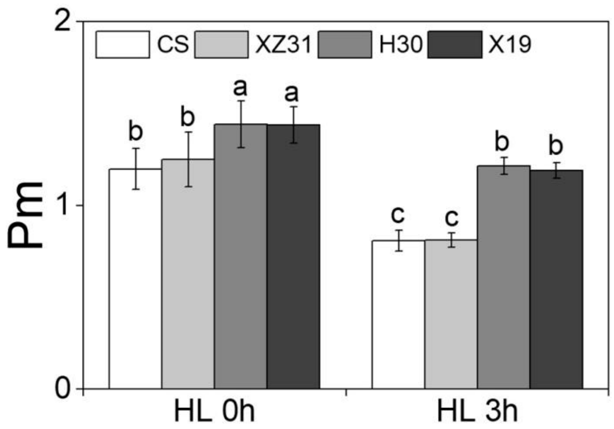

Figure S4. Effects of high light on maximum P700 signal (*P*m) in four Triticeae crops. The data represent means ± SD from three independent biological replicates ($n = 3$). Different lower-case letters indicate significant differences ($p < 0.05$) according to Duncan's multiplication range test. HL 0h and HL 3h represent high light for 0 h and 3 h, respectively.

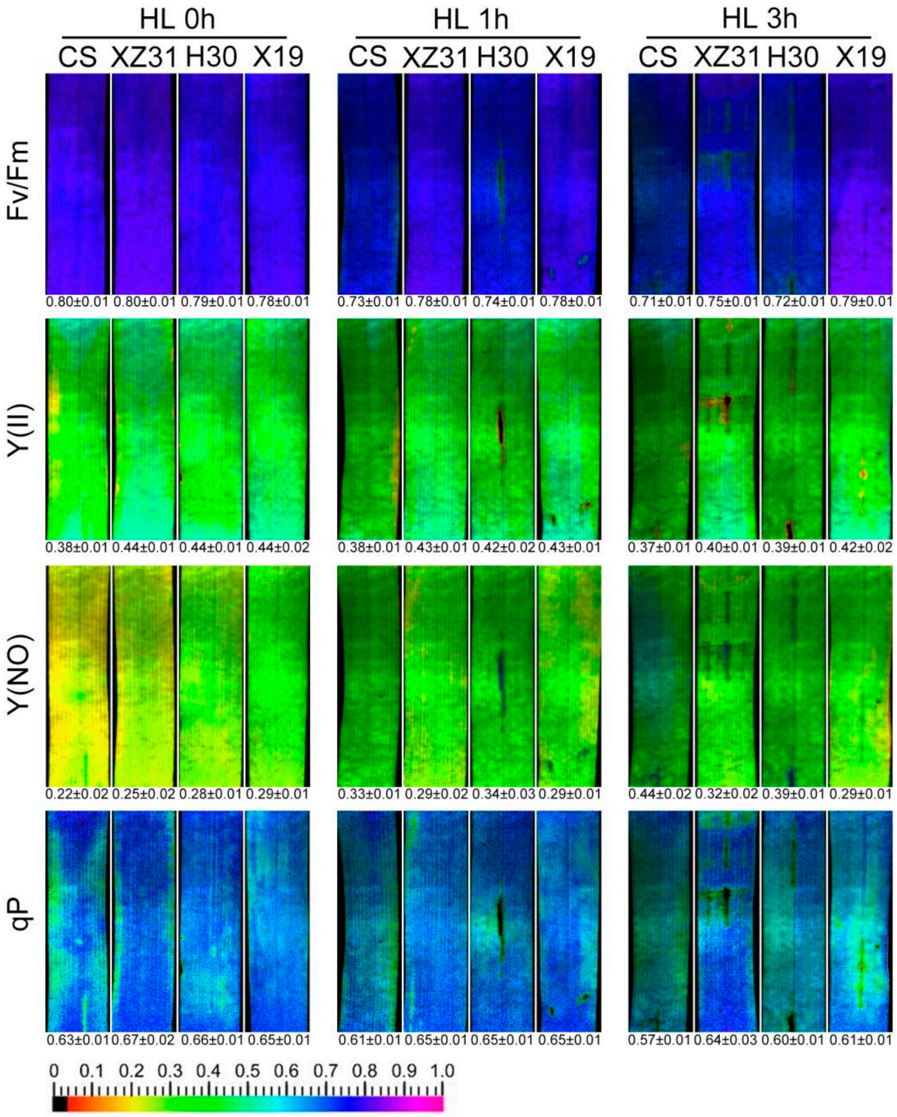

Figure S5. Effects of high light on chlorophyll fluorescence of four Triticeae crops. Fv/Fm, maximum efficiency of PSII photochemistry. Y(II), quantum yield of PSII electron transport. Y(NO), quantum yield of nonregulated energy dissipation. qP, photochemical quenching. The individual fluorescence images with quantitative values (± SD) are presented. HL 0h, HL 1h, and HL 3h represent high light for 0 h, 1 h, and 3 h, respectively.

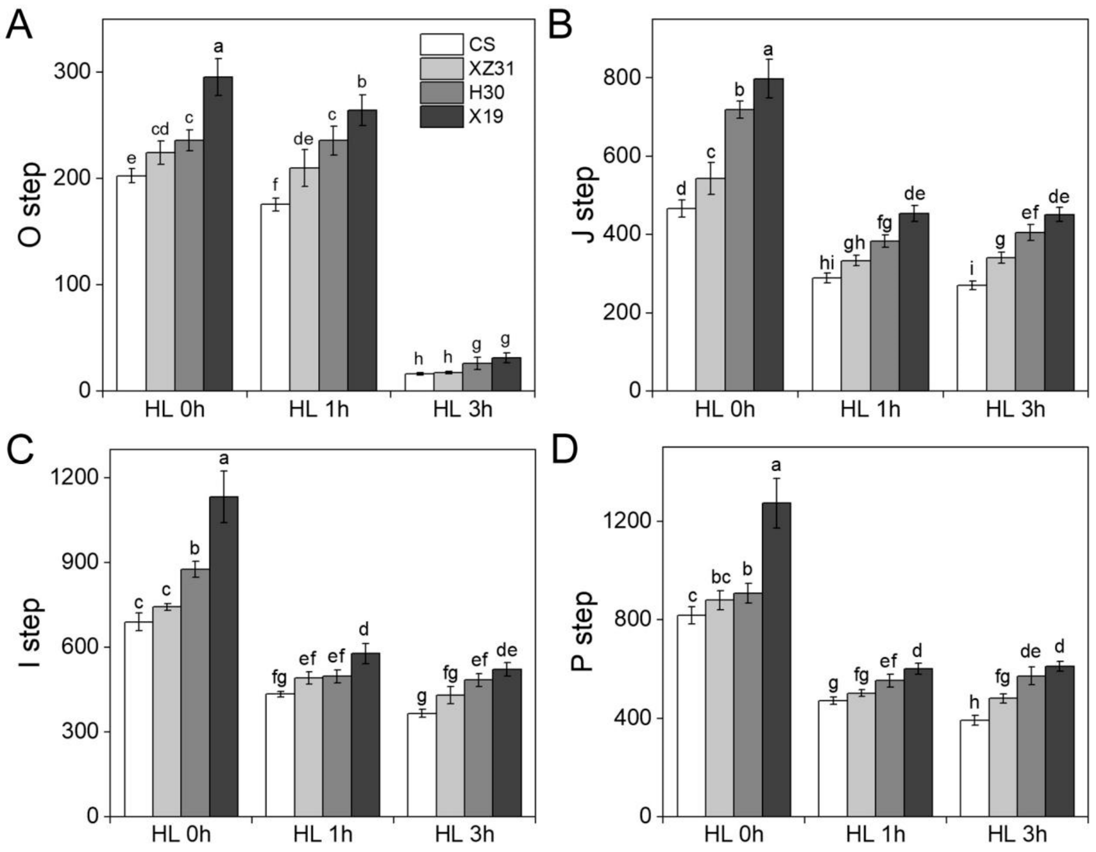

Figure S6. Effects of high light on the O-P steps of four Triticeae crops. Bars represent standard deviations from three independent biological replicates (*n* = 3). Different letters indicate significant differences ($p < 0.05$) according to Duncan's multiplication range test. HL 0h, HL 1h, and HL 3h represent high light for 0 h, 1 h, and 3 h, respectively.

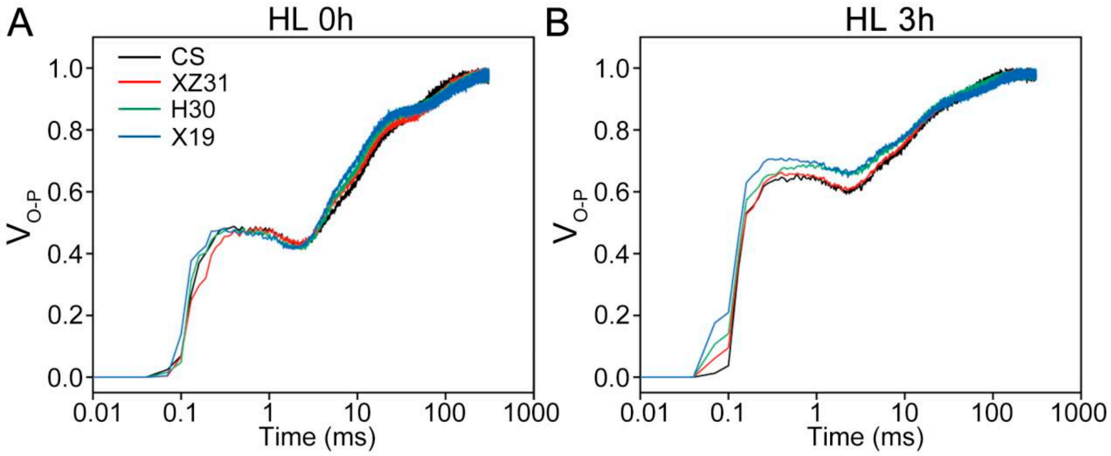

Figure S7. Effects of high light on $V_{\mathrm{O\text{-}P}}$ curves in four Triticeae crops. The data in the figure are from three replicated experiments (n = 3). HL 0h and HL 3h represent high light for 0 h and 3 h, respectively.

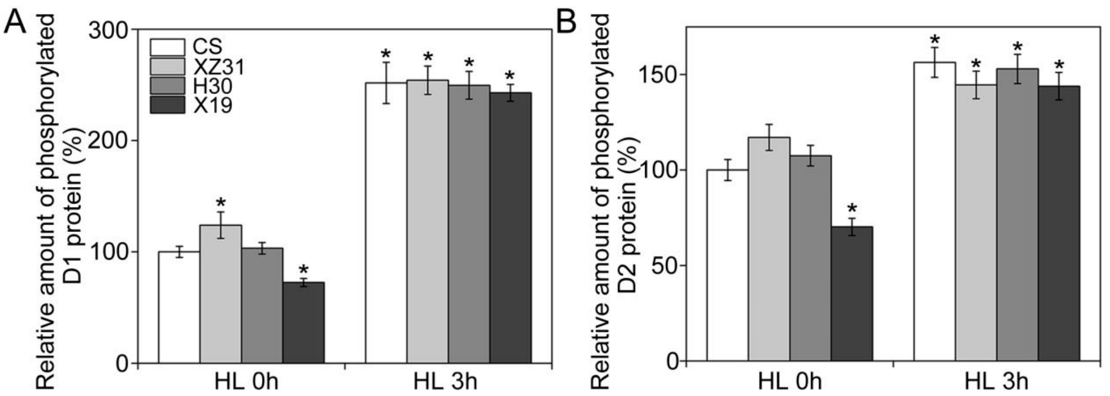

Figure S8. Quantitative data for thylakoid protein phosphorylation in four Triticeae crops under high light. (A) Relative level of phosphorylated D1 protein. (B) Relative level of phosphorylated D2 protein. The results are expressed relative to the amount of untreated CS (100%). * indicates a significant difference at $p < 0.05$ level. HL 0h and HL 3h represent high light for 0 h and 3 h, respectively.
